# Bronchotracheal Stenting Management by Rigid Bronchoscopy under Extracorporeal Membrane Oxygenation (ECMO) Support: 10 Years of Experience in a Tertiary Center

**DOI:** 10.1155/2021/8822591

**Published:** 2021-03-18

**Authors:** Sabrina Meyer, Anne-Sophie Dincq, Lionel Pirard, Sebahat Ocak, Jean-Paul D'Odémont, Philippe Eucher, Benoît Rondelet, André Gruslin, Laurie Putz

**Affiliations:** ^1^Department of Anesthesiology, Université Catholique de Louvain, CHU UCL Namur, Site Godinne, Yvoir, Belgium; ^2^Division of Pulmonology, Université Catholique de Louvain, CHU UCL Namur, Site Godinne, Yvoir, Belgium; ^3^Department of Cardiovascular Surgery and ECCP, Université Catholique de Louvain, CHU UCL Namur, Site Godinne, Yvoir, Belgium

## Abstract

**Purpose:**

Airway stenting offers good palliation and improves the quality of life in patients with inoperable bronchotracheal stenosis. However, in some cases, the management of stenting can be life-threatening. Hence, a strategy for maintaining oxygenation and hemodynamic stability should be anticipated to avoid critical situations. Herein, we report the use of extracorporeal membrane oxygenation (ECMO) in bronchotracheal stenting management to secure oxygenation and facilitate interventions.

**Methods:**

We retrospectively reviewed all patients who underwent rigid bronchoscopy under ECMO support for the management of bronchotracheal stenting at CHU UCL Namur hospital (Belgium), between January 2009 and December 2019.

**Results:**

We included 14 bronchoscopy cases performed on 11 patients (3 patients underwent 2 bronchoscopies) in this study; 12 were performed on males and 2 on females. The median age was 54 years. There were 11 benign and 3 malignant etiologies for the central airway obstruction/stenosis. Eight cases were supported by venovenous ECMO and six by venoarterial ECMO. The median ECMO time was 267 minutes. The weaning of ECMO support was successful in all cases. In most cases, the procedures were performed effectively and safely. Only two local complications caused by the cannulation of ECMO were reported, and anticoagulation was adapted to avoid bleeding at the operating site and clot formation in the system.

**Conclusion:**

Elective ECMO support was helpful and safe for the high-risk management of bronchotracheal stenting with rigid bronchoscopy and was not associated with any additional significant complications.

## 1. Introduction

Rigid bronchoscopy is a medical technique first described by Gustav Killian in 1898 and was used to remove a foreign body aspirated into the right main bronchus. It was abandoned in the second half of the 20^th^ century following the emergence of flexible bronchoscopy but regained importance with the growth of interventional pulmonology [1]. Rigid bronchoscopy has a lot of diagnostic and therapeutic indications. It is useful in endobronchial biopsies, extraction of foreign bodies, management of massive hemoptysis, and treatment of central airway obstructions [1]. Airway obstruction can cause significant morbidity and mortality. The etiology of airway obstruction could be malignant (primary tracheal or bronchial tumors, other thoracic tumors with direct extension into the airways, or extrathoracic tumors with thoracic metastases) or benign (autoimmune and infectious diseases, surgical anastomoses, postendotracheal intubation scars, chemical/thermal injuries, idiopathic tracheal stenoses, pseudotumors, etc.) [2]. Severe bronchotracheal obstruction or stenosis is commonly associated with respiratory distress. The treatment consists of debulking (with forceps; Argon, CO_2,_ and Nd : YAG lasers; electrocautery; cryotherapy; etc.), balloon dilatation, and/or stenting. In some cases, airway management for these procedures can be challenging [3]; in others, the procedure itself, especially airway stenting, can be dangerous. Indeed, secondary airway obstruction can occur due to poor deployment of the stent or due to bleeding, tumor breakdown, or airway perforation [4]. This can be particularly true for the removal of metallic tracheal stents [5].

Extracorporeal membrane oxygenation (ECMO) is a modality of extracorporeal life support. There are two main types of ECMO, namely venovenous ECMO (VV-ECMO) and venoarterial ECMO (VA-ECMO). In the VV-ECMO, blood is drained from the venous system and returned to the venous system. It allows gas exchange (oxygenation and removal of carbon dioxide) and is the treatment of choice in cases of respiratory failure with preserved cardiac function. In the VA-ECMO, blood is drained from the venous system and returned to the arterial system. It allows gas exchange and provides hemodynamic support [6, 7]. The ECMO also controls the patient's temperature with a heater-cooler system (to achieve normothermia in these patients). ECMO can be useful to secure oxygenation in severe cases of airway obstruction [8]. The purpose of this multiple case study was to describe the 10-year experience of a single European institution in providing ECMO support during high-risk rigid bronchoscopy for the management of bronchotracheal stenting and to determine its advantages and complications.

## 2. Materials and Methods

Medical charts of all patients over 18 years of age, who underwent rigid bronchoscopy under ECMO for the management of bronchotracheal stenting between 2009 and 2019 at CHU UCL Namur hospital (Belgium), were retrospectively reviewed in 2020. The local institutional review board approved this case report series (approval number: 11/2020). Written informed consent was not required as this was a retrospective study and the patients did not express any opposition to the use of their medical records for research purposes.

The data collected before and after rigid bronchoscopy included patient demographics, type and location of airway disease, reports of rigid bronchoscopy and anesthesia, ECMO settings, complications, and clinical outcomes. The data was extracted from the computerized records and detailed intraoperative patient protocols.

All patients included in this study had an initial central airway obstruction or stenosis with different etiologies diagnosed by flexible bronchoscopy. They were all complex cases and required bronchotracheal stenting management (including insertion, removal, or change of bronchotracheal stent). ECMO was indicated when the specialists felt that conventional intervention was risky or impossible and would put the patient's life in danger following uncertain intraoperative ventilation and oxygenation, given the risk of complete airway obstruction/perforation or massive bleeding.

Patient care was provided by a specialized multidisciplinary team involving anesthesiologists, pulmonologists, cardiovascular surgeons, perfusionists, and nurses. An intravenous line, an arterial line, and a central line were placed and continuous monitoring of oxygen saturation (pulse oximetry), blood pressure, ECG, and neuromuscular transmission and cerebral near-infrared spectroscopy were performed before the initiation of ECMO.

Depending on the patients' comorbidities and institutional habits, we either placed VV- or VA-ECMO. In our institution, ECMO support was set up in the operating room by cardiovascular surgeons prior to the procedures performed under local or regional anesthesia, sedation, or general anesthesia, depending on the patient's status and choice of the anesthesiologists and surgeons in the operating room. If ventilation or intubation was considered or known to be difficult, ECMO was systematically inserted and initiated under local anesthesia and maintained by a qualified perfusionist throughout the procedure. A blood test protocol at the ECMO circuit was followed and recorded in a database every 10 minutes. The patients' blood gases were analyzed regularly during the procedure.

Internal jugulo-femoral cannulation was used for VV-ECMO and double femoral cannulation for VA-ECMO in the majority of patients. Percutaneous cannulations were preferred in most cases at the surgeon's discretion, excluding VA-ECMO for which a surgical approach was performed for the femoral arterial cannula. We did not use peripheral cannulas for distal leg reperfusion in femoro-femoral ECMO because they were placed for a short period of time. We generally used 17–19 Fr arterial cannulas and 21–23 Fr venous cannulas (Bio-Medicus cannula; Medtronic, Minneapolis, MN, USA). The size of the cannula depends on the body mass index, vascular caliber, and the flow required to achieve adequate circulatory support. The ECMO device was a Stöckert Centrifugal Pump Console (LivaNova, PLC, London, EN, UK) combined with a Sorin Revolution Centrifugal pump (Sorin Group Italia Srl, Mirandola, MO, Italy). An X-ray confirmed that the cannulas were correctly positioned.

ECMO's anticoagulation management has evolved during the 10 years of the study. In all procedures, we used a heparin-coated ECMO circuit (Custom Tubing Pack with Bioline Coating, Maquet Getinge Group, Rastatt, Germany) and heparinized serum to purge the ECMO cannulas. During the first 3 years of the study, patients were anticoagulated with a bolus infusion of unfractionated heparin (50–100 UI/kg), the dose of which was gradually reduced over time to maintain the activated clotting time (ACT) within the therapeutic range (180–200 seconds). Subsequently, to reduce the risk of bleeding, anticoagulation therapy was discontinued. ECMO was initiated in normothermic conditions and the adequate flow was gradually established based on the theoretical flow calculated according to the body surface area and cardiac index.

Before or after ECMO placement, depending on the case, general anesthesia was induced using short-acting intravenous agents, such as propofol and remifentanil, and deep neuromuscular blockade using rocuronium. The Monsoon™ III Jet Ventilator (Acutronic Medical system AG, Hirzel, Switzerland) was fixed on the lateral entrance of the rigid bronchoscope (Efer-Dumon®) specially devoted to ventilation, and high-frequency jet ventilation (HFJV) was always provided in addition to ECMO support. Therapeutic rigid bronchoscopy was then performed by an interventional pulmonologist. Depending on the type of lesion, procedures such as balloon dilatation or ablative techniques of tissue destruction (laser, argon plasma coagulation, etc.) were performed before the insertion, removal, or change of bronchotracheal stents. In the absence of complications such as bleeding and respiratory or hemodynamic failure, patients were gradually weaned off the ECMO support at the end of the intervention. After the procedure, depending on their condition, the patients were either left intubated or extubated and then transferred to the intensive care unit for monitoring.

## 3. Results

Of the 658 rigid bronchoscopies performed during the study period, 14 (2.1%) were performed under ECMO for the management of bronchotracheal stenting and were included in the analysis (Table 1). These 14 bronchoscopies concerned 11 different patients (3 of them underwent 2 procedures). Of the 14 cases, 12 were performed in males and 2 in females. The median age was 54 years (range: 29–78 years). The etiologies of the initial airway pathologies were benign in 11 and malignant in 3 cases. The indications for rigid bronchoscopy were stent removal (three cases), stent insertion (four cases), or stent change (seven cases). The stents that needed to be changed or removed were all metallic. Depending on the cases, the new stents placed were fully covered metallic or silicone stents. The metallic stents used were Ultraflex stents (Boston Scientific, Natick, MA, USA) or Micro-Tech stents (Micro-Tech (Nanjing) Co., Ltd., China) and the silicone stents were Dumon™ stents (Novatech SA, La Ciotat, France).

All the patients underwent multiple surgeries, such as debulking, balloon dilatation, or stenting, for complex stenoses, prior to the procedure under ECMO. We used multiple severity criteria for the choice of ECMO placement in these cases. Some of these concerned patients, such as severely altered pulmonary function tests, particularly forced expiratory volume in 1 second (<49% of predicted value) and diffusing capacity of the lung for carbon monoxide (<40% of predicted value), oxygen-dependent patient, or patient with a single lung. The others concerned the procedure, such as localization of upper tracheal stenosis; presumption of the duration of the operative procedure to be more than 1 hour; stent failure in anterior rigid bronchoscopy due to hemodynamic, respiratory, or procedural causes; or severe stent damage resulting in tracheal stenosis (granulomas, free mesh of the stent in the tracheal lumen, and dislocation or deformity of the stent).

Eight cases were supported with VV-ECMO and six with VA-ECMO. We placed four ECMO devices under local anesthesia, two under regional anesthesia [9] (superficial cervical plexus block and transversus abdominis plane block), two under light sedation (with propofol or midazolam), and six under general anesthesia (three patients were sedated and intubated in the intensive care unit before the procedure). The median ECMO time was 267 minutes (range: 90–1740 minutes). Weaning of ECMO support was successful in 13 cases. In a single case, ECMO weaning was not successful immediately after the procedure due to poor oxygenation probably caused by significant endotracheal bleeding and was, therefore, removed the next day. The median ECMO time for patients directly weaned from ECMO support was 153 minutes (range: 90–274 minutes; Table 2).

In most cases, the procedure was safe. Nonetheless, two ECMO complications were observed: one patient developed an inguinal hematoma and another developed an inguinal lymphocele, which was drained after a month. The most frequent complications were due to the procedure itself; either stent removal was not possible, migration of the stent occurred after surgery, or the stenosis recurred early. We also reported one case of severe bronchospasm and four cases of tracheal bleeding, one of which required a transfusion of 2 units of red blood cells (RBCs). Four other blood transfusions (1 or 2 units of RBCs) were necessary for conditions other than hemorrhagic phenomena. The indication was a decrease in hemoglobin below the transfusion threshold, probably due to hemodilution caused by fluid priming (580–600 ml of crystalloid fluid) of the ECMO circuit in anemic patients preoperatively. The average preoperative hemoglobin of these four patients was 10.1 g/dL (range: 8–11.4 g/dL).

Following stent management by rigid bronchoscopy, seven patients were extubated in the operating room before transfer to the intensive care unit, five of whom went back to a hospital unit the next day, one after four days, and one was reintubated the day after the procedure and kept in the intensive care unit for 7 days. In the next seven cases of rigid bronchoscopy, all the patients were left intubated in the intensive care unit after the procedure and were extubated within 7 days. The average length of hospital stay was 7.2 days (range: 4–12 days). Of the 11 patients, 7 were alive at the time of data analysis in August 2020 and 4 died within a month after the procedure (range: 10–26 days).

## 4. Discussion

Airway stenting is a procedure proposed to patients with inoperable benign or malignant bronchotracheal stenosis or obstruction. It allows for rapid palliation, which can be life-saving and can also improve the quality of life [10]. There are different types of stents (silicone, uncovered metallic, and partially or fully covered metallic stents) with different characteristics, advantages, disadvantages, and indications. Depending on the type of stent used, their management (insertion, removal, or change) can sometimes be complicated, resulting in serious complications such as airway perforation, severe bleeding, reobstruction, and possibly death. The complications are so dangerous, especially with uncovered metallic stents [11, 12], that a strategy for maintaining oxygenation should be anticipated to avoid critical situations and has been extended to other difficult indications with covered stents in our experience.

Malpas et al. reviewed 45 cases of ECMO used in patients with central airway obstruction in 2018 and concluded that ECMO is an effective method of providing adequate oxygenation for the treatment of central airway obstruction with varied management [13]. Only 7 of these 45 cases involved a tracheal stent. The present study reports 14 additional successful cases of bronchotracheal stent management by rigid bronchoscopy under ECMO in a tertiary center.

We initially favored VV-ECMO, anticipating an oxygenation defect during the procedure. A few years later, we successfully used VA-ECMO for one patient with poor cardiac function and another who had not hemodynamically endured the previous procedure. After these conclusive experiences, we favored VA-ECMO for all patients during the last few years of the retrospective review. It has various advantages: it avoids jugular cannulations in patients with fragile access caused by multiple catheterizations, ensures good hemodynamic conditions, and helps the operator obtain easier cervical movements during the surgery/procedure and an extended head position, without any risk to the cannulas. The indication of the type of ECMO must be discussed on a case-by-case basis. The use of ECMO can be associated with a lot of complications [6]. In this report, there were only two local inguinal complications with VA-ECMO and no neurologic thromboembolic complications such as leg ischemia, renal failure, gastroenteric hemorrhage, or intracranial hemorrhage. This is explained by the fact that ECMO remained in place for a short period of time [8], either small doses of or no anticoagulants were used, and normothermic conditions were maintained.

We reported four cases of airway bleeding, which could have been due to the heparin used during ECMO. As our experience progressed, we reduced the dose of unfractionated heparin used to maintain ACT within the therapeutic range of 180–200 seconds and eventually discontinued the intravenous heparin. We noticed a decreased extended bleeding at the operating site during the study period without anticoagulation therapy [8, 13]. Therefore, as described in other publications [14], we also avoid intravenous anticoagulation. We only used a heparin-coated ECMO circuit and heparinized serum to purge the ECMO cannulas.

In this case series, we have reported only a few complications specific to the use of ECMO in bronchotracheal stenting. The most frequent complications were due to the procedure itself, such as nonoptimal stent placement, stent migration, or stent obstruction. These complications occurred early after the stent placement and were frequently reported in the literature (40–60%) [15]. In our series, four procedures (28%) led to complications within the first 7 days of stent insertion and one stent could not be removed as expected. Of the 11 patients, 4 died within a month after the procedure. Three of these had malignant airway obstruction and underwent an emergency tracheal stenting for severe dyspnea in an advanced oncological context. The fourth patient died unexpectedly of a cardiac arrest of undetermined origin at his home 10 days after the procedure.

The difficult cases of bronchotracheal stenting can lead to severe complications and even a fatal outcome. This is particularly true when the management concerns the removal/change of metallic stents [5]. This risk is obviously serious for the patient but also stressful for the healthcare team because the situation may suddenly become critical. Since ECMO provides oxygenation throughout the operation, the patient is protected against hypoxia, and the interventional pulmonologist has enough time to think about the best strategies to safely restore the airway in real time with less stress [4]. The opinions of the pulmonologists in our institution are positive; they work in a less stressful environment and are efficient in handling critical cases like these. In addition, the ECMO implemented preventively could be useful in cases wherein the oxygenation conditions at the end of the intervention are insufficient, as observed in one of our patients in whom ECMO weaning was not successful immediately after the procedure and was, therefore, removed the next day, when the oxygenation was improved.

The role of an anesthesiologist is also particularly important. The anesthesiologist works in collaboration with the perfusionist and pulmonologist and ensures optimal surgical conditions and maintains an adequate respiratory and hemodynamic status of the patient. In our case series, we used HFJV through the rigid bronchoscope. This specific technique of ventilation allows airway sharing between anesthesiologists and pulmonologists in open airway systems, thereby providing excellent working conditions for the operator. In our case series, we systematically added jet ventilation to the ECMO for each patient, which helped in the reduction of atelectasis. In cases of VA-ECMO, there may be a flow competition between the retrograde reinjection of oxygenated blood through the peripheral VA-ECMO and the ejection of hypoxemic blood by the heart if the systolic function is sufficient. Therefore, the jet ventilation ensures good oxygenation of blood after the pulmonary circulation and avoids differential hypoxemia between the upper body and lower body [16].

Our case review has several limitations. One of them is the retrospective, nonrandomized, and uncontrolled methodology. This can result in multiple biases of selection, misclassification, and information. However, it seems difficult and unethical to perform a prospective, randomized, and/or double-blinded trial in patients with life-threatening conditions. This is also probably why literature only includes case reports/series and retrospective studies in this regard. Another limitation is the limited sample size in our study. More cases are needed to confirm our results and determine specific criteria for selecting patients who would benefit from ECMO during their bronchoscopic procedures.

## 5. Conclusions

While HFJV is sufficient for most patients undergoing rigid bronchoscopic bronchotracheal stent management, we report the successful anticipatory use of ECMO support (VV- or VA-ECMO) for rare high-risk patients in whom ventilation management was uncertain during the procedure due to the risk of complete airway obstruction/perforation or massive bleeding. The indication of ECMO for the procedure was discussed by multidisciplinary teams according to multiple criteria based on computed tomography or flexible bronchoscopy findings, the characteristic and position of the stent, and the patient's comorbidities. The use of ECMO with minimal anticoagulation was not associated with additional significant complications and ensured blood oxygenation and hemodynamic stability.

## Figures and Tables

**Table 1:** Patient characteristics.

Case	Age (years)	Sex	Cause of airway obstruction	Intervention	Initial stent	New stent

1	29	M	Postintubation tracheal stenosis	Stent removal	Fully covered metallic stent	—
2	39	M	Postintubation tracheal stenosis	Stent change	Fully covered metallic stent	Silicone stent
3	55	M	Postintubation tracheal stenosis	Stent change	Fully covered metallic stent	Fully covered metallic stent
4	56	M	Postintubation tracheal stenosis	Stent change	Fully covered metallic stent + silicone stent	Silicone stent
5	68	M	Postintubation tracheal stenosis	Stent change	Fully covered metallic stent	— (Withdrawal failed)
6	54	F	Postintubation tracheal stenosis	Stent removal	Partially covered metallic stent	—
7	60	M	Postintubation tracheal stenosis	Stent removal	Partially covered metallic stent	—
8	49	M	Postintubation tracheal stenosis	Stent insertion	—	Silicone stent
9	78	M	Extrinsic tracheal compression by a thyroid carcinoma	Stent insertion	—	Fully covered metallic stent
10	38	M	Bronchial obstruction by metastasis from a testicular cancer	Stent insertion	—	Fully covered metallic stent
11	49	F	Tracheobronchial obstruction by a primary lung cancer	Stent insertion	—	Fully covered metallic stent
12	59	M	Postpneumonectomy tracheobronchial stenosis	Stent change	Fully covered metallic stent	Fully covered metallic stent
13	59	M	Postpneumonectomy tracheobronchial stenosis	Stent change	Fully covered metallic stent	Fully covered metallic stent
14	62	M	Stenosis due to lung transplant sutures	Stent change	Fully covered metallic stent	Fully covered metallic stent

M : male; F : female.

**Table 2:** ECMO characteristics.

Case	Type of ECMO	Run time (minutes)	Weaning	Survival (>60 days)
1	VV	180	OR	Alive
2	VA	274	OR	Alive
3	VV	121	OR	Alive
4	VV	1740	ICU	Alive
5	VV	125	OR	Alive
6	VV	115	OR	Alive
7	VV	90	OR	Alive
8	VV	135	OR	Alive
9	VA	140	OR	Dead
10	VV	209	OR	Dead
11	VA	100	OR	Dead
12	VA	116	OR	Alive
13	VA	134	OR	Dead
14	VA	255	OR	Alive

VV: venovenous; VA: venoarterial; OR : operating room; ICU : intensive care unit.

## Data Availability

Data can be made available upon request to the corresponding author.
